# Translocation of a Polymer through a Crowded Channel under Electrical Force

**DOI:** 10.1155/2017/5267185

**Published:** 2017-03-26

**Authors:** Tingting Sun, Yunxin Gen, Hujun Xie, Zhouting Jiang, Zhiyong Yang

**Affiliations:** ^1^Department of Physics, School of Information and Electronic Engineering, Zhejiang Gongshang University, Hangzhou 310018, China; ^2^Department of Applied Chemistry, School of Food Science and Biotechnology, Zhejiang Gongshang University, Hangzhou 310018, China; ^3^Department of Physics, China Jiliang University, Hangzhou 310018, China; ^4^Department of Physics, Jiangxi Agricultural University, Nanchang, Jiangxi 330045, China

## Abstract

The translocation of a polymer chain through a crowded cylindrical channel is studied using the Langevin dynamics simulations. The influences of the field strength *F*, the chain length *N*, and the crowding extent ρ on the translocation time are evaluated, respectively. Scaling relation τ ~ *F*^−α^ is observed. With the crowding extent ρ increasing, the scaling exponent α becomes large. It is found that, for noncrowded channel, translocation probability drops when the field strength becomes large. However, for high-crowded channel, it is the opposite. Moreover, the translocation time and the average translocation time for all segments both have exponential growth with the crowding extent. The investigation of shape factor 〈δ〉 shows maximum value with increasing of the number of segments outside *s*. At last, the number of segments inside channel *N*_in_ in the process of translocation is calculated and a peak is observed. All the information from the study may benefit protein translocation.

## 1. Introduction

Translocation of a variety of biopolymers through channels or pores plays an important role in biological systems [1], for example, injection of DNA from a virus to bacteria [2], protein transport through membrane channels [3], translocation of DNA and RNA across nuclear pores [4], and translocation of nascent proteins inside the ribosomal tunnel or across the endoplasmic reticulum [5–8]. In addition, the translocation process is also useful in the range of biotechnological applications, such as rapid DNA sequencing [9, 10], gene therapy, drug delivery, and drug discovery [11]. A large number of experimental [12–17], theoretical [18–23], and simulation studies [24–37] focus on polymer translocation.

Kasianowicz et al. [12] demonstrated that single-stranded DNA and RNA molecules can be driven through the water-filled  *α*-hemolysin channel under electric field. And the passage of each molecule is signaled by a blockade in the channel current. Improving this technique, the nucleotide sequence of DNA or RNA can be read off. In addition, solid-state nanopores have been used instead of the  *α*-hemolysin channel in other experiments [13–17]. The scaling relationship of translocation time with chain length is *τ* ∝ *N*^1.27^, which is different from that obtained by Kasianowicz et al., *τ* ∝ *N*.

These experimental results have promoted many theoretical [18–23] and computational studies [24–36]. Muthukumar treated the translocation of polymer chains with classical nucleation theory where the nucleation rate *k*_0_ is assumed to be a constant for each monomer. Luo et al. studied the translocation of polymer based on the fluctuating bond (FB) and Langevin dynamics (LD) models with the bead-spring approach. The coarse-grained model in terms of the multiple particles collision (MPC) method was also applied in the study of polymer translocation.

In actual fact, the effect of crowding on the translocation dynamics should not have been neglected, because in the cell cytoplasm crowding due to macromolecular species and structural obstacles can be as high as 50% by volume. Moreover, in biological processes, protein-protein interactions are fundamentally important [37]. And this crowding environment has considerable influence on reaction rates, protein folding rates, and equilibria in vivo.

In this paper, we investigate the dynamics of polymer translocation through a crowded channel. And the extent of crowding is considered. Our model and the simulation technique are described in Section 2. Simulation results and discussion are presented in Section 3 and Section 4 is a conclusion.

## 2. Method of Calculation

In the simulations, we use the Langevin equation to study the Brownian motion of particles where the equation of motion for each bead at position *r*_*i*_ is described as

(1) $$\begin{array}{c} m{\ddot{r}}_{i}\left(\;t\;\right)=-\nabla U_{i}-\xi {\dot{r}}_{i}\left(\;t\;\right)+F+W_{i}\left(\;t\;\right), \end{array}$$

where *m* is the bead mass, *ξ* is the friction coefficient, *F* denotes the external force due to the applied voltage represented by $F=F\hat{z}$, and *W*_*i*_(*t*) is the random force which satisfies the fluctuation-dissipation relation [38]

(2) Wit=0,Wit·Wjt′=6kBTξδijδt−t′.

The total interaction is as follows:

(3) $$\begin{array}{c} U_{i}=U_{FENE}^{i}+\Sigma U_{LJ}^{ij}. \end{array}$$

The finitely extensible nonlinear elastic (FENE) [39] spring potential interaction between two successive beads is

(4) $$\begin{array}{c} U_{FENE}\left(\;r_{ij}\;\right)=-\frac{1}{2}kR_{0}^{2}\ln\left(\;1-\frac{{r_{ij}}^{2}}{R_{0}^{2}}\;\right), \end{array}$$

where *k* is the spring constant, *R*_0_ is the maximum allowed separation between connected monomers, and here *r*_*ij*_ is the distance between consecutive monomers.

And a repulsive Lennard-Jones (LJ) potential is applied between all bead pairs considering both excluded volume and Van der Waals interactions between beads:

(5) ULJrij=4εσrij12−σrij6+ε,rij≤21/6σ,0,rij>21/6σ.

Here, *σ* is the diameter of a bead, *ε* is the depth of the potential, and *r*_*ij*_ is the distance between two beads.

**Figure 1:**
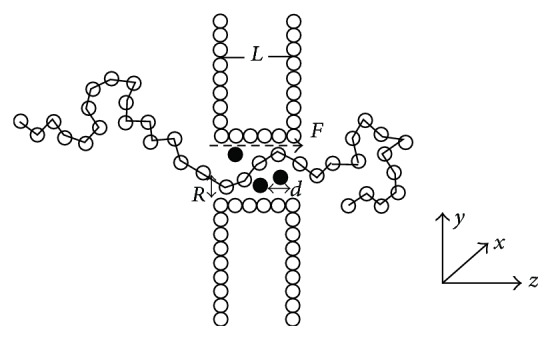
A schematic illustration of a polymer moving through a crowded channel. The channel length is *L*. The channel radius is *R*. The external electric force *F* is applied in the *z*-axis direction inside the channel. The black spherical obstacles represent crowding environments. And the diameter of the obstacle is *d*.

The model of translocation is illustrated in Figure 1.

A voltage is applied across the pore. The wall is formed by columns of stationary particles. Between the bead-wall particle pairs and bead-pore particle pairs, there exists the same short range repulsive LJ interaction as described in (5). Crowding is modeled by randomly distributed spherical obstacles. The diameter of the obstacles is *d*. And the interactions between obstacles and polymers or other particles are all described as (5). The volume of an obstacle is *V*_*o*_ = (4/3)*π*(*d*/2)^2^, and the volume of the channel is *V*_*c*_ = *πR*^2^ · *L*. We can describe the density of the obstacles in the channel *ρ* = *N*_*o*_*V*_*o*_/*V*_*c*_. Here *N*_*o*_ is the number of obstacles distributed inside the channel. In this work, the diameter *σ* and the LJ interaction strength  *ε*  fit the length units and the system energy. The time scale is *t*_LJ_ = (*mσ*^2^/*ε*)^1/2^. The parameters are *σ* = 1, *R*_0_ = 2*σ*, *k* = 7*ε*, *ξ* = 0.7, and *k*_*B*_*T* = 1.2*ε* [30]. In the simulations, *L* = 5, *R* = 2, and *d* = 1. Firstly, the first monomer of the polymer is placed in the entrance of the channel, and the remaining monomers are to obtain an equilibrium configuration undergoing thermal collisions described by the Langevin thermostat. Then, the first monomer is released, and under the external electrical force the polymer begins to cross through the channel. The translocation time is defined as the time interval between the first monomer in the channel and the last monomer out of the channel. 2000 independent runs are averaged in our simulations.

## 3. Results and Discussion

### 3.1. Translocation Time

**Figure 2:**
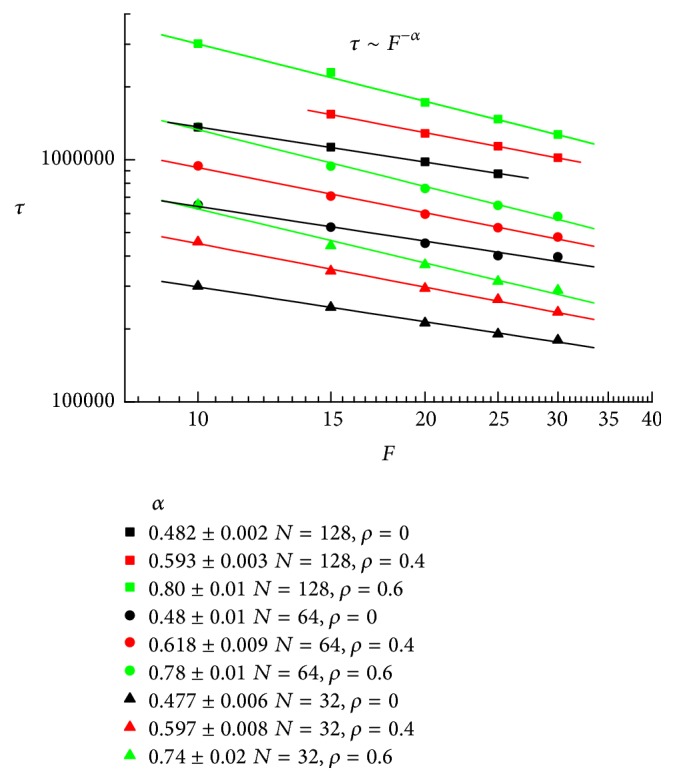
Translocation time *τ* as a function of the external electric force *F* for different crowding extent *ρ* and chain length *N*.

As a result of external electric force, the polymer can overcome the entropic barrier due to the loss of the number of the configurations during translocation. It is obvious that the translocation time decreases with increasing electric force. In Figure 2, we fit the translocation time on the external force and find the scaling relation *τ* ~ *F*^−*α*^.

The exponent *α* is 0.482 ± 0.002, 0.48 ± 0.01, and 0.477 ± 0.006 without crowding for *N* = 128, 64, and 32, respectively. The same scaling behavior has been found using DPD simulation [40]. And the exponent is 0.48 ± 0.01, which is completely in accord with our results. However, no crowding environment is discussed in that work. From the figure, it is certainly found that as the crowding extent *ρ* increases, the translocation time *τ* increases under the same channel and chain. That is because the more crowded the channel, the larger the entropic barrier existing during translocation. However, as the crowding extent *ρ* increases, the scaling exponent *α* also increases. The value of *α* is 0.593 ± 0.003, 0.618 ± 0.009, and 0.597 ± 0.008 under *ρ* = 0.4 for *N* = 128, 64, and 32. This is due to the increased entropic resistance as *ρ* increases. For *ρ* = 0.6, the exponent *α* apparently becomes larger. For *N* = 128, 64, and 32, the value of *α* is 0.80 ± 0.01, 0.78 ± 0.01, and 0.74 ± 0.02, respectively. It is shown that the dependence of the translocation time on the external force is stronger for longer chain under *ρ* = 0.6, while the scaling exponent *α* for different chain length *N* is nearly unobvious under *ρ* = 0 and *ρ*⁡ = 0.4. That means the influence of chain length to the scaling relation only happens under large crowded channel.

**Figure 3:**
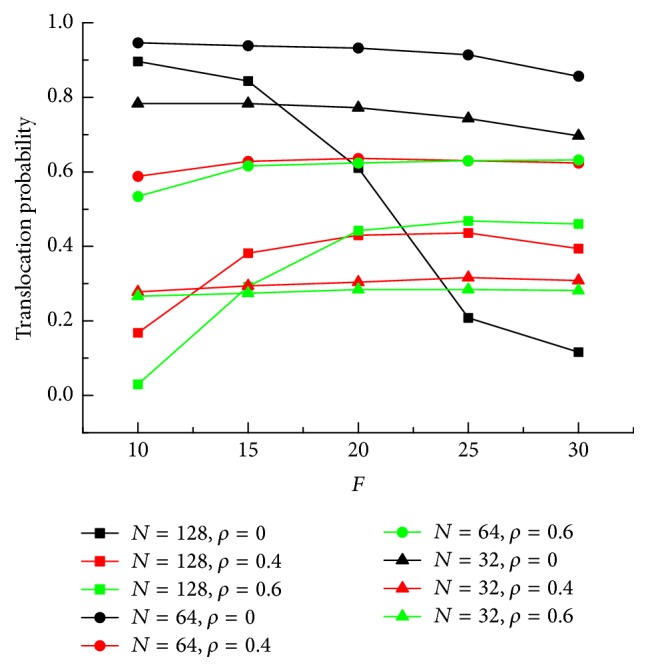
Translocation probability as a function of the external electric force *F* for different crowding extent *ρ* and chain length *N*.

In Figure 3, we show the translocation probability of successful events as a function of the external electric force *F* for different crowding extent *ρ* and chain length *N*. First, when *ρ* = 0, the translocation probability decreases with increasing translocation force *F*. For short chain *N* = 64 and 32, the change is slow. However, for *N* = 128, the translocation probability is above 80% under *F* = 10 and 15. Because, under weak electric force, the translocation velocity is relatively slow, the monomers already translocated can diffuse and let the rest of chain through the channel. However, the translocation probability quickly drops to 60% and 20% for *F* = 20 and 25. And it is only 10% when *F* = 30. This is due to the fact that, with increasing *F*, the monomers inside channel will get larger acceleration speed and exit crowding happens easily. That causes the translocation probability drops. Above all, it shows that, for *ρ* = 0, low electric field leads to more successful translocation. Considering the crowded extent of the channel, *ρ* = 0.4 and 0.6 are both investigated. It shows that, for *N* = 32 and 64, the translocation probability changes little with *F* increasing. The value is around 30% and 60%, respectively. However, for longer chain, *N* = 128, when *F* = 10, the translocation probability is 17%, and it increases to 40% when *F* changes to 15. The phenomenon is more obvious for *ρ* = 0.6. We focus on the results of *N* = 128. The translocation probability is only 3% under *F* = 10. With increasing *F*, translocation probability increases first and then approaches saturation when *F* > 20. It is quite different from that of *ρ* = 0. It can be well understood that, with increasing *ρ*, entropic barrier for the chain translocation becomes greater; thus, the chain will be dragged back out of the channel if the electric field is weak, which led to the low success of translocation probability. On the contrary, with the electric force *F* increasing, the chain's backward motion out of the channel will effectively be prohibited, and the translocation probability increases.

**Figure 4:**
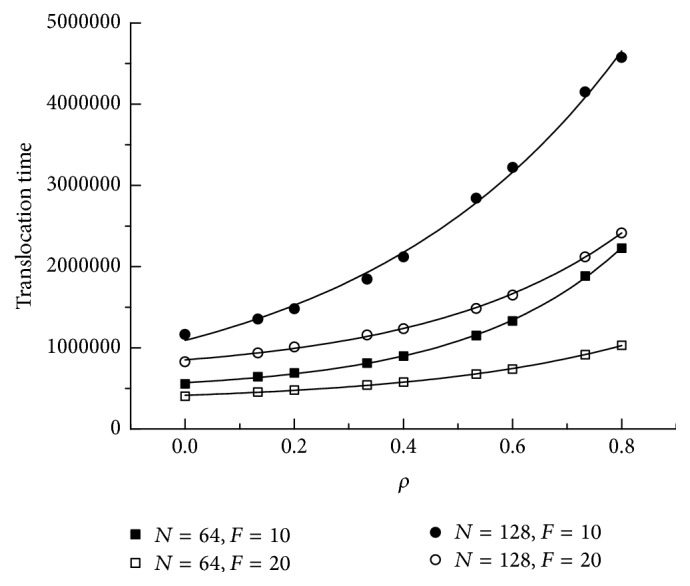
Translocation time as a function of the crowding extent *ρ* for different external force *F* and chain length *N*.

Figure 4 shows the translocation time as a function of the crowding extent *ρ*. We find that translocation time increases with increasing *ρ*. It keeps appropriate exponential growth. As is shown, the translocation time of *F* = 10 is higher than that of *F* = 20. For the same length of polymer, with crowding extent increasing, the interval between *F* = 10 and *F* = 20 becomes larger.

**Figure 5:**
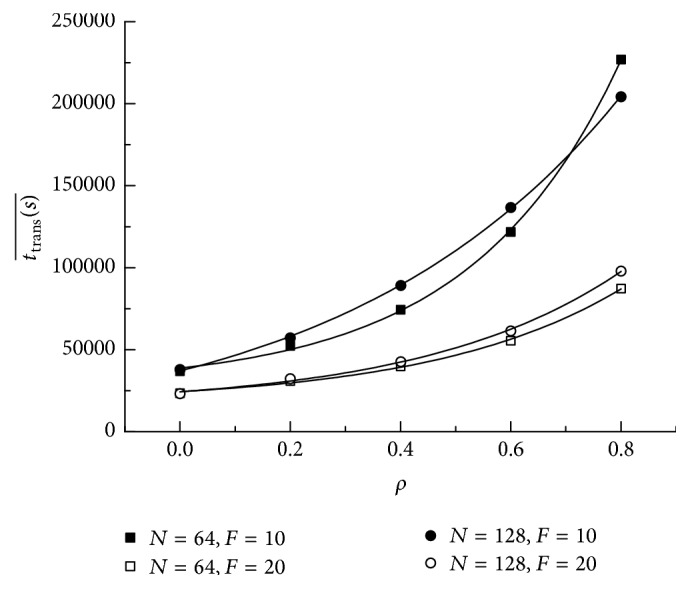
Average translocation time for all segments as a function of the crowding extent *ρ*.

Figure 5 demonstrates average translocation time for all segments under different crowding extent *ρ*. It is observed that the average time also has exponential increase. For larger electric field *F* = 20, the interval between *N* = 64 and *N* = 128 becomes larger with the crowding extent increasing. However, for smaller *F* = 10, a strange phenomenon appears. The interval between *N* = 64 and *N* = 128 increases and then decreases. Before *ρ* = 0.7, the average translocation time of segments of *N* = 128 is longer than that of *N* = 64. When *ρ* = 0.7, the average time reaches the same value. As *ρ* = 0.8, the average translocation time of segments of *N* = 64 is longer than that of *N* = 128. The following can be interpreted: when the channel is much crowded, with the addition of weak electric field *F* = 10, the longer chain is easier to adjust its conformation outside to let the rest of the monomers pass through the channel.

**Figure 6:**
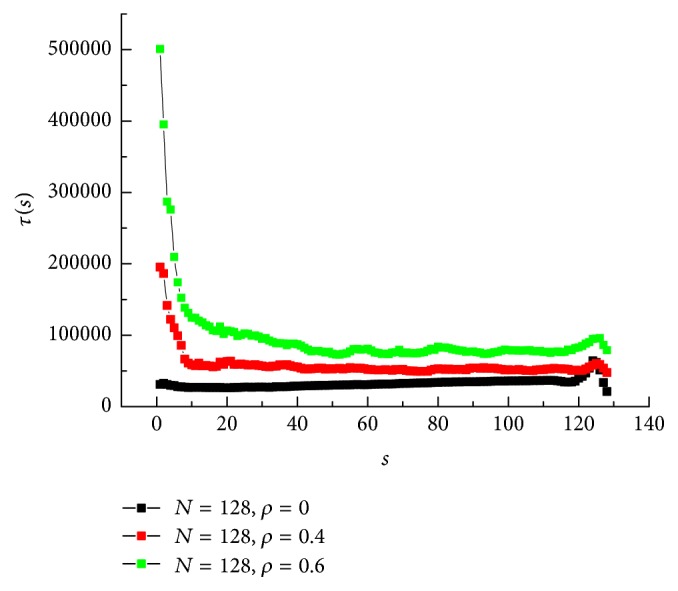
Translocation time of bead *s*  *τ*(*s*) under different crowding extent *ρ* for chain length *N* = 128 and electric force *F* = 15.

Figure 6 shows the translocation time of bead *s*  *τ*(*s*) for *N* = 128 and crowding extent *ρ* = 0, 0.4, and 0.6. For *ρ* = 0.6, the translocation time of *s* = 1 is the largest. The reason is that the crowding channel will prevent the chain entering into it, so a high entropy barrier exists for the first several monomers. This can interpret why the translocation probability of *ρ* = 0.6 is smaller than *ρ* = 0 in Figure 3. Behind quick decreasing, *τ*(*s*) decreases slowly and approaches saturation. For the last several monomers, a short peak is shown. This is because the channel exit will be crowded with translocated monomers. For less crowding channel *ρ* = 0.4, almost a similar trend is investigated. However, the saturation stage is longer. When the chain goes through the noncrowded channel, the translocation time is almost unchanged till *s* = 120. It seems that the translocation is easier than crowded channel. Also a peak is shown at the end of the curve. The reason is the same as above.

### 3.2. Chain Size and Conformations

The instantaneous shape of an individual configuration may be described by several ratios based on the principal components *L*_1_^2^ ≤ *L*_2_^2^ ≤ *L*_3_^2^ of *S*^2^ = *L*_1_^2^ + *L*_2_^2^ + *L*_3_^2^, that is, the orthogonal components of the squared radius of gyration taken along the principal axes of inertia [41, 42]. 〈*δ*〉 is obtained by combining the reduced components of *S*^2^ to a single quantity that varies between 0 (sphere) and 1 (rod) [43, 44]

(6) $$\begin{array}{c} \langle \;\delta \;\rangle =1-3\langle \;\frac{L_{1}^{2}L_{2}^{2}+L_{2}^{2}L_{3}^{2}+L_{3}^{2}L_{1}^{2}}{{\left(L_{1}^{2}+L_{2}^{2}+L_{3}^{2}\right)}^{2}}\;\rangle . \end{array}$$

**Figure 7:**
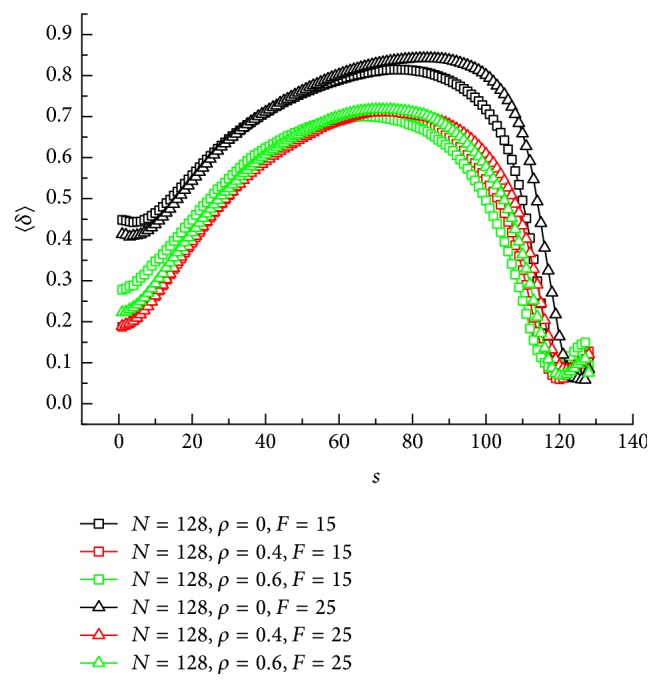
A plot of average shape factor 〈*δ*〉 versus the number of segments out of channel *s* under different crowding extent *ρ* and external electric force *F* for chain length *N* = 128.

In Figure 7 we show average shape factor 〈*δ*〉 versus the number of segments out of channel *s*. Different crowding extent *ρ* and electric force *F* are both considered. As shown in Figure 7, with the increasing of *s*, 〈*δ*〉 shows a maximum with increasing *s*. It represents that the shape of chain is most spread as 〈*δ*〉 reaches a maximum value. For *ρ* = 0, the value of *s* corresponding to the maximum of 〈*δ*〉 is larger than that of *ρ* = 0.4 and 0.6. That means the incompact conformation in the translocation for *ρ* = 0 appears later than crowded channel. Another finding is when 〈*δ*〉 goes through the maximum, it drops more quickly for noncrowded channel than crowded channel.

**Figure 8:**
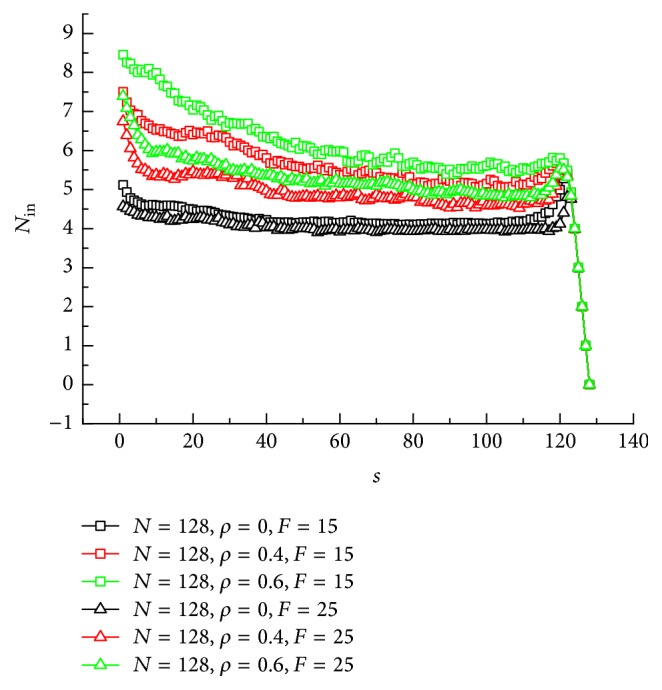
Number of segments inside channel *N*_in_ versus the number of segments out of channel *s*.

The number of segments inside channel *N*_in_ versus the number of segments out of channel *s* has been shown in Figure 8 for *N* = 128, *ρ* = 0, 0.4, and 0.6, and *F* = 15 and 25. There is a peak that existed in each curve at *s* = 120~125. When there is no crowded particle in the channel, it is shown that *N*_in_ decreases slightly and has a long plateau till *s* = 120. It seems that the translocation through noncrowded channel is smooth. At last, *N*_in_ increases to a peak value and then it drops. The peak implies that the translocation velocity decreases at the last lap of translocation. It is due to the fact that translocated segments will cause the exit to be crowded. However, if the channel is crowded with many particles occupied, here *ρ* = 0.4 and 0.6 are both considered. The value of *N*_in_ decreases relatively greater than *ρ* = 0. It can be explained that the crowding particles inside of the channel will push the chain out of the channel. It leads to the translocation velocity increasing; thus *N*_in_ decreases. Another finding is that the peak of the curve for electric force *F* = 15 is less clear than that for *F* = 25. This is because larger electric force will accelerate the crowding nearby the exit. And some segment will enter into the channel to ensure a successful translocation.

## 4. Conclusions

Using the Langevin dynamics simulations, we investigate the translocation of a polymer chain through a crowded cylindrical channel. We first observe a scaling relation *τ* ~ *F*^−*α*^ between the translocation times *τ* on the external force *F*. The scaling exponent *α* increases as the crowding extent becomes larger. As an example, for *N* = 128, the scaling exponent *α* is 0.482 ± 0.002, 0.593 ± 0.003, and 0.80 ± 0.01 for *ρ* = 0, 0.4, and 0.6, respectively. This shows that the more crowded the channel, the much larger the entropic barrier during translocation. The translocation probability under different field strength is also investigated. The translocation probability decreases when the field strength becomes large for noncrowded channel. However, for high-crowded channel, it is the opposite. At the same time, we find that the translocation time has an exponential growth with the crowding extent *ρ*. Average translocation time for all segments as a function of the crowding extent *ρ* has a similar law. Considering the translocation time of each segment under different crowding extent, the peak at the end of translocation shows the crowding of translocated segments near the exit. On the other hand, the shape factor 〈*δ*〉 is studied to give the shape change of chain during the translocation. With the number of segments outside increasing, a maximum value 〈*δ*〉 is shown. Behind the maximum value, 〈*δ*〉 decreases more quickly for noncrowded channel than that for crowded channel. At last, the number of segments inside channel *N*_in_ in the process of translocation is investigated. A peak existing at the end of translocation means the translocation velocity decreases at the last lap of translocation. These findings will shed light on the translocation dynamics of polymer.

## References

[1] Lodish H., Baltimore D., Berk A., Zipursky S. L., Matsudaira P., Darnell J. (1995). *Molecular Cell Biology, Scientific*.

[2] Laemmli U. K., Favre M. (1973). Maturation of the head of bacteriophage T4: I. DNA packaging events. *Journal of Molecular Biology*.

[3] Darnell J. E., Lodish H., Baltimore D. (1990). *Molecular Cell Biology*.

[4] Alberts B., Bray D., Lewis J., Raff M., Roberts K., Watson J. Molecular Biology of the Cell (New York: Garland, 1994), An enormous but excellent textbook which puts the reader in touch with current ideas without too much pain on the way. Molecular genetics is covered in the context of the life of the cell.

[5] Lingappa V. R., Chaidez J., Yost C. S., Hedgpeth J. (1984). Determinants for protein localization: *β*-lactamase signal sequence directs globin across microsomal membranes. *Proceedings of the National Academy of Sciences of the United States of America*.

[6] Choi K. M., Brimacombe R. (1998). The path of the growing peptide chain through the 23S rRNA in the 50S ribosomal subunit; a comparative cross-linking study with three different peptide families. *Nucleic Acids Research*.

[7] Gabashvili I. S., Gregory S. T., Valle M. (2001). The polypeptide tunnel system in the ribosome and its gating in erythromycin resistance mutants of L4 and L22. *Molecular Cell*.

[8] Hardesty B., Kramer G. (2000). Folding of a nascent peptide on the ribosome. *Progress in Nucleic Acid Research and Molecular Biology*.

[9] Han J., Turner S. W., Craighead H. G. (1999). Entropic trapping and escape of long DNA molecules at submicron size constriction. *Physical Review Letters*.

[10] Turner S. W. P., Cabodi M., Craighead H. G. (2002). Confinement-induced entropic recoil of single DNA molecules in a nanofluidic structure. *Physical Review Letters*.

[11] Ji Z., Su J., Liu C., Wang H., Huang D., Zhou X. (2014). Integrating genomics and proteomics data to predict drug effects using binary linear programming. *PLoS ONE*.

[12] Kasianowicz J. J., Brandin E., Branton D., Deamer D. W. (1996). Characterization of individual polynucleotide molecules using a membrane channel. *Proceedings of the National Academy of Sciences of the United States of America*.

[13] Meller A. (2003). Dynamics of polynucleotide transport through nanometre-scale pores. *Journal of Physics: Condensed Matter*.

[14] Li J., Stein D., McMullan C., Branton D., Aziz M. J., Golovchenko J. A. (2001). Ion-beam sculpting at nanometre length scales. *Nature*.

[15] Li J., Gershow M., Stein D., Brandin E., Golovchenko J. A. (2003). DNA molecules and configurations in a solid-state nanopore microscope. *Nature Materials*.

[16] Storm A. J., Storm C., Chen J., Zandbergen H., Joanny J.-F., Dekker C. (2005). Fast DNA translocation through a solid-state nanopore. *Nano Letters*.

[17] Muthukumar M. (1999). Polymer translocation through a hole. *The Journal of Chemical Physics*.

[18] Ghosal S. (2006). Electrophoresis of a polyelectrolyte through a nanopore. *Physical Review E*.

[19] Dubbeldam J. L. A., Milchev A., Rostiashvili V. G., Vilgis T. A. (2007). Polymer translocation through a nanopore: a showcase of anomalous diffusion. *Physical Review E*.

[20] Wong C. T. A., Muthukumar M. (2007). Polymer capture by electro-osmotic flow of oppositely charged nanopores. *The Journal of Chemical Physics*.

[21] Gopinathan A., Kim Y. W. (2007). Polymer translocation in crowded environments. *Physical Review Letters*.

[22] Wong C. T. A., Muthukumar M. (2008). Polymer translocation through a cylindrical channel. *The Journal of Chemical Physics*.

[23] Luo K., Ala-Nissila T., Ying S.-C. (2006). Polymer translocation through a nanopore: a two-dimensional Monte Carlo study. *Journal of Chemical Physics*.

[24] Luo K., Huopaniemi I., Ala-Nissila T., Ying S.-C. (2006). Polymer translocation through a nanopore under an applied external field. *Journal of Chemical Physics*.

[25] Huopaniemi I., Luo K., Ala-Nissila T., Ying S.-C. (2006). Langevin dynamics simulations of polymer translocation through nanopores. *The Journal of Chemical Physics*.

[26] Forrey C., Muthukumar M. (2007). Langevin dynamics simulations of ds-DNA translocation through synthetic nanopores. *The Journal of Chemical Physics*.

[27] Luo K., Ala-Nissila T., Ying S.-C., Bhattacharya A. (2007). Influence of polymer-pore interactions on translocation. *Physical Review Letters*.

[28] Luo K., Ala-Nissila T., Ying S.-C., Bhattacharya A. (2007). Heteropolymer translocation through nanopores. *The Journal of Chemical Physics*.

[29] Luo K., Ala-Nissila T., Ying S.-C., Bhattacharya A. (2008). Sequence dependence of DNA translocation through a nanopore. *Physical Review Letters*.

[30] Luo K., Ala-Nissila T., Ying S.-C., Bhattacharya A. (2008). Dynamics of DNA translocation through an attractive nanopore. *Physical Review E*.

[31] Luo K., Ala-Nissila T., Ying S.-C., Bhattacharya A. (2008). Translocation dynamics with attractive nanopore-polymer interactions. *Physical Review E*.

[32] Gauthier M. G., Slater G. W. (2008). A Monte Carlo algorithm to study polymer translocation through nanopores. II. Scaling laws. *The Journal of Chemical Physics*.

[33] Gauthier M. G., Slater G. W. (2008). Sequence effects on the forced translocation of heteropolymers through a small channel. *The Journal of Chemical Physics*.

[34] Izmitli A., Schwartz D. C., Graham M. D., De Pablo J. J. (2008). The effect of hydrodynamic interactions on the dynamics of DNA translocation through pores. *The Journal of Chemical Physics*.

[35] Chen J.-X., Zhu J.-X., Ma Y.-Q., Cao J.-S. (2014). Translocation of a forced polymer chain through a crowded channel. *Europhysics Letters*.

[36] You Z.-H., Li J., Gao X. (2015). Detecting protein-protein interactions with a novel matrix-based protein sequence representation and support vector machines. *BioMed Research International*.

[37] Yang Z., Li S., Zhang L., Ur Rehman A., Liang H. (2010). Translocation of *α*-helix chains through a nanopore. *The Journal of Chemical Physics*.

[38] Allen M., Tildesley D. (1987). *Computer Simulation of Liquids*.

[39] Grest G. S., Kremer K. (1986). Molecular dynamics simulation for polymers in the presence of a heat bath. *Physical Review A*.

[40] He Y.-D., Qian H.-J., Lu Z.-Y., Li Z.-S. (2007). Polymer translocation through a nanopore in mesoscopic simulations. *Polymer*.

[41] Chang H., Venkatesan B. M., Iqbal S. M. (2006). DNA counterion current and saturation examined by a MEMS-based solid state nanopore sensor. *Biomedical Microdevices*.

[42] Sun T., Zhang L. (2004). Effect of secondary structure on the conformations and folding behaviors of protein-like chains. *Polymer*.

[43] Sun T., Zhang L. (2005). Conformations and dynamics of adsorbed protein-like chains. *Polymer*.

[44] Jagodzinski O., Eisenriegler E., Kremer K. (1992). Universal shape properties of open and closed polymer chains: renormalization group analysis and Monte Carlo experiments. *Journal de Physique I*.

